# Irisin, Exercise, and COVID-19

**DOI:** 10.3389/fendo.2022.879066

**Published:** 2022-06-17

**Authors:** Hugo Rodrigues Alves, Guilherme Schittine Bezerra Lomba, Cassiano Felippe Gonçalves-de-Albuquerque, Patricia Burth

**Affiliations:** ^1^ Department of Cell and Molecular Biology, Fluminense Federal University, Niterói, Brazil; ^2^ Laboratory of Immunopharmacology, Federal University of the State of Rio de Janeiro, Rio de Janeiro, Brazil; ^3^ Postgraduate Program in Biotechnology, Fluminense Federal University, Rio de Janeiro, Brazil

**Keywords:** COVID-19, irisin, exercise, inflammation, adipomyokine

## Abstract

Muscle and adipose tissue produce irisin during exercise. Irisin is thermogenic adipomyokine, improves glucose and lipid metabolism, and ameliorates the effects of obesity-driven inflammation, metabolic syndrome, and diabetes. In addition, exercise-induced irisin activates anti-inflammatory pathways and may play an essential role in improving the outcomes of inflammatory conditions, such as coronavirus disease (COVID-19). COVID-19 infection can activate different intracellular receptors and modulate various pathways during the course of the disease. The cytokine release storm (CRS) produced is significant because it promotes the context for systemic inflammation, which increases the risk of mortality in patients with severe acute respiratory syndrome coronavirus 2 (SARS-CoV2). In addition, viral infection and the resulting organ damage may stimulate the mitogen-activated protein kinase(MAPK) and toll-like receptor 4 (TLR4)/toll interleukin receptor (TIR)-domain-containing adaptor (MyD88) pathways while negatively modulating the AMP-activated protein kinase (AMPK) pathway, leading to increased inflammatory cytokine production. Exercise-induced irisin may counteract this inflammatory modulation by decreasing cytokine production. Consequently, increased irisin levels, as found in healthy patients, may favor a better prognosis in patients with SARS-CoV2. This review aims to explore the molecular mechanisms underlying the anti-inflammatory properties of irisin in mitigating CRS and preventing severe outcomes due to infection with SARS-CoV2.

## Introduction

In 2012, a group of researchers reported the discovery of irisin, a previously uncharacterized hormone derived from the transmembrane protein fibronectin type III domain-containing protein 5 (FNDC5), which is widely expressed in numerous tissues (1). Based on the functional characterization of irisin, it may be defined as a myokine (1) and an adipokine released during exercise (2). Further research has found that irisin mediates the conversion of white adipose tissue to brown adipose tissue, resulting in a thermogenic effect (1). In addition, irisin improves glucose and lipid metabolism (3–5) and ameliorates the effects of obesity-driven inflammation, metabolic syndrome, and diabetes (6–8). As a result, several beneficial effects on human health derived from exercise, such as reduced inflammation in pathological states, may be ascribed in part to irisin secretion (9, 10). Given these positive effects of exercise-induced irisin, it may play a role in mitigating the effects of other conditions that trigger systemic inflammation.

Coronavirus disease 2019 (COVID-19) is a highly infectious disease caused by severe acute respiratory syndrome coronavirus 2 (SARS-CoV2), which has reached alarming proportions during the pandemic (11). Its pathological features include exacerbated inflammation and induction of a cytokine storm, which contribute to the development of severe immune reactions (12–14). Therefore, one of the main therapeutic strategies targets the hyperactivation of immune responses (12). Multiple studies hypothesize exercise to be a protective factor against COVID-19 through the downregulation of the cytokine storm and reduction of sequelae (15, 16). It can also mitigate the progress of the infection, improve immune response and even work as a coadjutant therapy for COVID-19 (17). In this way, one observational study was able to associate moderate levels of exercise with a lower prevalence of COVID-19-related hospitalization (18). However, the role of irisin as a potential mechanism mediating exercise’s benefits in COVID-19 outcomes has yet to be uncovered.

In this review, we aim to discuss the anti-inflammatory effects of irisin, focusing on the MAPK, AMPK, and TLR4/MyD88 intracellular pathways, and examine how exercise-induced irisin may improve the outcomes of inflammatory conditions, such as COVID-19.

## Irisin

Irisin has 112 amino acid residues and is a proteolytic cleavage subproduct of the FNDC5 protein (1). FNDC5 is a type 1 membrane protein that contains 209 amino acids in mice and 212 in humans⁠. The FNDC5 precursor has five domains: the signal sequence (29 aa), fibronectin type III (94 aa), functionally unidentified region (28 aa), transmembrane (19 aa), and cytoplasmic (39 aa) domains. The N-terminal signal sequence is located in the extracellular environment, and the cytoplasmic domain is located at the C-terminus (1, 19, 20).

⁠Irisin has a very similar structure to fibronectin type III (FNIII) domains: a β-sandwich domain with four β-strands on one side and three on the other (19). However, irisin forms continuous antiparallel eight-stranded β-sheet dimers, a feature rarely found in other FNIII domains. Thus, irisin dimerization is associated with structural stability, receptor binding, and triggering signaling cascades (19).⁠

In rodents, irisin was identified in different tissues: muscle, lung, liver pancreas, adrenal glands, kidneys, and even in the central nervous system (21, 22). In humans, *FNDC5* is predominantly expressed in muscle tissue, and its expression patterns establish a few primary predictors of circulating irisin, such as muscle mass and age (23). Therefore, increased muscle tissue due to a resistance training program could be a way to increase circulating irisin levels, and these observations are substantiated by studies showing an increase in circulating irisin levels after aerobic and resistance exercise sessions (24, 25). Furthermore, lower circulating irisin levels in older people have been noted because human muscle tissue mass decreases annually after 50 years of age (26).

Because older adults (> 60 years old) have lower physiological irisin levels, their circulating irisin levels tend to increase at a higher rate than in younger adults after training sessions, with a higher increase noted after intense training (24). Although exercise frequency is positively associated with increased irisin blood levels (25) populations that frequently exercise did not receive equal benefits from training interventions (23, 24). Thus, further research is necessary to understand the relationship between exercise and circulating irisin levels in trained and untrained populations. Interestingly, participants with a greater body mass index were less influenced by the acute irisin response due to exercise, a condition termed “irisin resistance syndrome” (25). Therefore, subjects who showed lower body mass index (BMI) levels throughout the intervention also experienced notable increases in circulating irisin levels (24).

Both irisin and its precursor are N-glycosylated proteins (1)⁠. In FNDC5, two asparagines (Asn36 and Asn81) were identified as the glycosylation sites. The modification is associated with critical functions such as cellular stability and localization of FNDC5. In addition, glycosylation increases the half-life and membrane incorporation of the protein. In contrast, unglycosylated FNDC5 has a shorter half-life and is abundantly retained in the endoplasmic reticulum (27)⁠.

Irisin has two additional potentially glycosylated asparagine residues (Asn7 and Asn52). Glycosylation may stimulate irisin secretion because FNDC5 membrane incorporation depends on it. In addition, adipose tissue browning is intensified with glycosylated irisin, and glycosylation does not impact irisin dimerization (19, 27, 28).

The mechanisms associated with FNDC5 cleavage into irisin and its release are still uncertain, but a disintegrin and metalloproteinase (ADAM) family member are purportedly involved. ADAM family inhibitors caused irisin levels to decrease, even though FNDC5 expression did not change (29). The first characterized binding partners for irisin were found in adipocytes and osteocytes, and irisin was identified as a potential modulator of bone metabolism (30). In binding assays, integrins such as αV/β5, and to a lesser extent α1/β1, possess a high affinity for irisin. Furthermore, the expression of integrin-like signaling mediators increased with irisin stimulation. Conversely, irisin signaling decreased with the use of integrin inhibitors. Thus, the proposed receptors for irisin in tissues are the αV-integrin family of proteins (30, 31)⁠.

Irisin may act through an alternative pathway in the lungs of mice with ischemia/reperfusion-induced injury. Irisin may enter injured cells, protecting them by targeting the mitochondrial uncoupling protein 2 (UCP2), reducing cellular oxidative stress. Remarkably, this effect appears to be mediated by lipid raft endocytosis of irisin from the bloodstream, indicating that irisin may act regardless of the presence of the receptor (32).

FNDC5 and irisin expression are regulated by the transcriptional coactivator peroxisome proliferator-activated receptor-gamma coactivator 1 alpha (PGC1-α). An increase in PGC1-α expression in the muscle of transgenic mice is associated with the browning of white adipose tissue (WAT) in a process mediated by irisin (1). Additionally, PGC1-α is associated with exercise-mediated benefits and energy metabolism, leading to the hypothesis that exercise may stimulate irisin release (1)

PGC1-α interacts with several transcription factors to regulate FNDC5 synthesis during exercise. In particular, the transcription factors estrogen-related receptor alpha (ERRα) and cAMP response element-binding protein (CREB) are two presumed PGC1-α partners that increase FNDC5 synthesis (33, 34)⁠. Furthermore, nuclear hormone receptors such as glucocorticoid receptor (GR), constitutive androstane receptor (CAR), and farnesoid X receptor (FXR) also increase FNDC5 mRNA levels in cell cultures. Notably, cortisol can modulate FNDC5/irisin transcription in some human hepatoma cell lines (35–37).⁠ Because cortisol suppresses inflammation, we asked whether irisin could function alongside cortisol as an anti-inflammatory myokine in response to high-intensity exercise or inflammation.

Further, mothers against decapentaplegic homolog *3* (SMAD3) reduced FNDC5/irisin and PGC1-α expression because SMAD3 ablated mice showed increased serum irisin and high PGC1-α and FNDC5 expression in muscle. Additionally, TGF-β signaling activates SMAD3 signaling and blocks FNDC5 and PGC1-α transcription (38)⁠.

## COVID-19

In 2020, the world witnessed the rise of a new infectious disease caused by viral pneumonia, named COVID-19. Disease outbreaks began in 2019 in Wuhan, China, and spread to other countries and continents (39). As a result, the World Health Organization declared the disease a public health emergency in January 2020 and a pandemic in March 2020. As the pandemic progressed, over 400 million cases of COVID-19 and over 5.5 million deaths have been reported worldwide (11). Although a few infected individuals remain healthy and asymptomatic, the clinical spectrum of COVID-19 varies greatly, with increasing severity found in groups presenting comorbidities, including obesity, hypertension, diabetes, cardiovascular diseases, and old age (40, 41). The main clinical symptoms include fever, cough, respiratory distress/pneumonia, headache, anosmia, and ageusia, although atypical symptoms may also occur (39, 42).

SARS-CoV-2 causes the COVID-19 (42–44). It is a single-stranded RNA virus capable of infecting humans and other mammals, and it promotes disease due to the virus’s features and interaction with the host’s immune system (45). The interaction of the viral capsid protein S (spike protein) and human receptor proteins, mainly ACE-2 (angiotensin-converting enzyme 2) and TMPRSS2 (transmembrane protease, serine 2) enable virus entry into host cells (46). Indeed, ACE-2 levels are correlated with SARS-CoV-2 invasiveness potential and tropism in specific tissues. Tissues and organs expressing ACE-2, such as the lungs, heart, adipose tissue, and gastrointestinal tract, are the preferred targets for viral invasion (39, 47).

After viral uptake and fusion, its genetic material is uncoated and expressed, resulting in viral life cycle progression and replication (46). In the replication state, few or no symptoms are prevalent because tissue damage and immune responses are minimal, whereas organ damage and increasing severity of symptoms may occur as the infection progresses (14, 48). With immune cell recruitment, cytokine production begins to occur during immune response exacerbation. The expression of several types of cytokines may be increased in patients with COVID-19, such as IL1β, IL1RA, IL6, IL7, IL8, IL9, IL10, IFNγ, and TNFα (49, 50). Hence, one of the hallmarks of COVID-19 pathophysiology, hyperimmune activation, leads to a cytokine storm that is directly linked to disease severity (51). Although considerable research has been done, the COVID-19 literature evolves with new data released, and further research will continue while the pandemic persists.

## COVID-19-Related Inflammatory Pathways Modulated by Irisin

### AMP-Activated Protein Kinase Pathway

Studies have investigated the intracellular signaling cascades involved in irisin activity. For example, one study associated the AMPK/mTOR signaling pathway with irisin’s reported effects such as improved glucose uptake by muscle cells, reduced inflammation and insulin resistance, reduced blood pressure, conversion of WAT to brown adipose tissue (BAT), promotion of autophagy, and inhibition of pancreatic cancer cell growth (52). Herein, we will focus on the signaling pathways involved in the modulation of immune responses, leading to reduced inflammation and cytokine release. Furthermore, we hypothesized that irisin treatment could mitigate the effects of the cytokine storm in patients with severe COVID-19 by modulating the AMPK pathway (53).

The AMPK pathway is activated in response to an increase in adenosine monophosphate (AMP) concentration which can result from several circumstances, such as exercise, decreased adenosine triphosphate (ATP) production, and activation by crosstalk with other pathways. AMPK activation leads to the phosphorylation of intracellular targets, regulating glucose and lipid metabolism, cell growth, gene transcription, and other functions such as mTOR activation blockage, which is an intracellular pathway linked to irisin signaling (54). Furthermore, integrin αV/β5 signaling may be involved in irisin-mediated AMPK activation (55), and the pathway is associated with the protective role of irisin against cellular damage/cytokines, in addition to suppressing inflammation (56). Exercise activates AMPK pathway. However, the effect magnitude will depend on exercise intensity, duration, glycogen availability, and training status (57). Interestingly, high-intensity training did not improve AMPK activation in hypertensive rats, which contrasts with low- and medium-intensity exercise (58).

Xiong et al. (2018) described the effects of FNDC5/irisin on inflammation and macrophage function in mouse adipose tissues. FNDC5 ablation resulted in inflammation, AMPK inhibition, and M1 polarization. The administration of FNDC5 and its overexpression alleviated these effects. M1 macrophages support inflammatory responses through proinflammatory cytokine secretion of factors such as tumor necrosis factor-α, and interleukin-1β (59). Macrophage dysregulation is observed in chronic inflammatory diseases, such as obesity, and the induction of M1 polarization triggers cytokine release, impairing adipose tissue function (60). Obesity and other chronic proinflammatory conditions are COVID-19 comorbidities that influence disease severity. Therefore, avoiding M1 polarization *via* irisin-AMPK pathway activation could protect against COVID-19 disease development.

The AMPK and mTOR signaling pathways are intimately related, with opposite effects on immune system regulation (61). Irisin activates AMPK and blocks the mTOR pathway (29), resulting in decreased NF-κB activity and a consequent decrease in innate and adaptive immune responses (62, 63). Therefore, these pathways were linked to COVID-19 hyper inflammation and may serve as a potential therapeutic target (64), and the anti-inflammatory effects of irisin may result due to AMPK/mTOR regulation.

### Mitogen-Activated Protein Kinase Pathway

The MAPK pathway is extensively studied in association with irisin, comprising p38 and extracellular signal-regulated kinase (ERK)-mediated signaling. These pathways are related to several beneficial effects such as adipose tissue browning, glucose uptake, decreased insulin resistance, bone cell proliferation, and neural differentiation (52).

The activation of p38 MAPK induces cytokine release in individuals with COVID-19 (13, 65, 66). In addition, MAPKs are upregulated under several proinflammatory conditions and induce IL-6, IL-1β, and TNF-α secretion (67). Remarkably, irisin reduced inflammation and alleviated lung injury and acute respiratory distress syndrome in mice by decreasing the levels of the same cytokines released through MAPK pathway activation (68). Furthermore, irisin treatment decreased p38 and NF-κB activation induced by lipopolysaccharide (LPS) (68). Zhang et al. (2016) also reported anti-inflammatory effects of irisin treatment in treatment in mice for atherosclerosis, and found that IL-6 expression was reduced by p38 MAPK/NF-κB suppression (69).

Curiously, exercise triggers MAPK and NF-κB signaling pathways (70–75), and both promote proinflammatory states (67). P38 MAPK is an essential component in the cellular stress response that may act in physiological and pathological conditions, such as exercise and inflammatory states (67, 73). In addition, nonesterified fatty acid concentration may increase during exercise, stimulating TLR4/MAPK pathway, with no effect on NF-kB (73). Conversely, exercise may downregulate MAPK and NF-kB pathways. For instance, aerobic exercise attenuated proinflammatory macrophage polarization while decreased NF-kB and MAPK activation in LPS-exposed mice (76). Also, decreased p-p38 levels in rats’ lung inflammation submitted to aerobic exercise-trained (77). In addition, resistance training decreased MAPK activation after exercise (78, 79). Different aspects will change MAPK levels during and after exercise, such as age and exercise modality (80). The effects of irisin and exercise on MAPK/NF-kB are still controversial. However, a recent study sheds light on this debate: exercise-induced irisin counteracted inflammatory states by disturbing MD2-TLR4 complex formation and inhibiting MAPK and NF-kB pathways activation (81).

### Toll-Like Receptor 4/MyD88 Pathway

Toll-like receptors (TLRs) in immune cells recognize pathogen-associated molecular patterns (PAMPs) or danger-associated molecular patterns (DAMPs) (82). For example, TLR4 recognizes LPS present on the wall of Gram-negative bacteria, whereas viral dsDNA activates TLR3 (83, 84). In addition, the Toll interleukin receptor (TIR)-domain-containing adaptor (MyD88) is part of the TLR signaling pathway in all TLR family members and triggers inflammatory responses (84). However, TLRs are known to act differently. For instance, TLR3 and TLR4 activate MyD88-independent signaling pathways to induce proinflammatory responses, whereas cells from MyD88-knockout mice are unresponsive to TLR7 activation (84, 85).

Another signaling pathway reportedly modulated by irisin and related to the regulation of inflammatory responses is the TLR4/MyD88 pathway (86, 87). High concentrations of irisin in mice decreased TLR4 and MyD88 mRNA levels, consequently reducing NF-κB activation (86, 87). In this context, a decrease in proinflammatory cytokine secretion for IL-1β, IL-6, and TNF-α in macrophages was observed (86). Studies have shown that irisin can reduce brain ischemic/reperfusion injury and the proinflammatory activity of macrophages (86, 87). Hence, through the negative modulation of the TLR4/MyD88 pathway, irisin could improve the condition of patients with COVID-19 and their outcomes.

With the progression of SARS-CoV2 infection, a CRS may occur, which is significantly associated with the mortality of patients with COVID-19 (13). CRS is generated by the release of viral components in infected cells, leading to the uncontrolled activation of inflammatory signaling pathways (88). CRS causes oxidative stress, disseminated intravascular coagulation, severe metabolic acidosis, and multi-organ failure, inducing “viral sepsis” (13).

The second stage of injury, induced by CRS during influenza infections, can be mediated by DAMP-TLR4 dependent activation that results in pulmonary injury (89). We hypothesized that similar events occur during SARS-CoV2 infection, in which DAMP released during the second stage of SARS-CoV2 infection can further damage the lungs (88–90). Therefore, as irisin reduces TLR4 expression levels, which could decrease patient immune responses to PAMPs and DAMPs, and maybe COVID-19 patient prognosis. A similar therapeutic approach has been proposed to enhance adenosine signaling and inhibit the TLR/NF-κB pathway, diminishing the associated adverse effects (88). Additionally, the SARS-CoV2 spike protein can activate TLR4 and induce IL-1β production (91). Therefore, irisin effects may be extended, potentially acting on the prevention of patient conditions deterioration and interfering with macrophage viral recognition.

Obesity is a comorbidity of SARSCoV2 infection, resulting in a poor prognosis that implicates other signaling pathways. First, the TLR4 pathway, which is activated by saturated fatty acids, is one of the primary triggers for obesity-induced inflammation (92–95). Second, obese patients show increased levels of fatty acids in the serum, making them susceptible to systemic non-esterified fatty acid (NEFA) cytotoxicity which is responsible for increased oxidative stress and tissue damage (93). In addition, NEFA cytotoxicity may be further accentuated in obese patients with severe COVID-19 due to decreased albumin levels, contributing to more free fatty acids in serum because these are transported in the plasma by albumin (93, 96). In addition to obesity, hypertension also is clinical comorbidity associated with higher mortality risk in COVID-19 patients (97). In hypertension, the renin-angiotensin-aldosterone system (98, 99) and LPS-TLR4-MyD88 pathway are activated (100–102) and might be a propensity to inflammatory events during SARS-CoV-2 infection. Also, proinflammatory cytokines may trigger TLR4/MyD88/NF-kB pathways activation, contributing to hypertensive response in the hypothalamus (103). Considering exercise effects on hypertension, low- and medium-intensity exercise effectively improved blood pressure through TLR4/NF-kB suppression (58) and improved hypertension in rats (103). Exercise modulates TLR4 and NF-kB expressions, depending on the training intensity: acute, moderate, low, and on the training frequency: acute and regular (104). Acute training could be potentially inflammatory, stimulating an increase in TLR4 levels on the cell surface (104, 105); while resistance training presents an anti-inflammatory pattern, leading to MyD88 and TLR4 downregulation of mRNA expression and protein levels (106, 107). Moderate regular exercise has anti-inflammatory properties, downregulating TLR4 and MyD88 (108–110). TLR4 downregulation by irisin could play a prominent role in preventing or decreasing hyperinflammation in obese and hypertensive patients, and healthy individuals who undergo regular moderate exercise. Thus, we suggest that activation of the TLR4 pathway in patients with COVID-19 may be linked to poor patient outcomes (3, 111).

*Pseudomonas aeruginosa* is the most common bacterial coinfection in patients with COVID-19 (112). *P. aeruginosa* is a Gram-negative bacterium associated with severe hospital-acquired infections and is a fundamental cause of sepsis (Ramachandran, 2014; Shafran et al., 2021). Irisin can ameliorate inflammation in mice with LPS-induced lung injury (68), which may be critical for patients with COVID-19 because they are prone to contracting bacterial infections during hospitalization (90, 112). Thus, irisin may prevent secondary Gram-negative bacterial immune response exacerbation and sepsis.

Besides regulating the activation of inflammatory pathways, irisin can modulate gene transcription and may potentially reduce the transcriptional levels of several genes related to COVID-19 (113). For example, irisin decreased the expression of *TLR3*, which is upregulated in COVID-19 patients, which prevented the hyperactivation of innate immunity. TLR3 ablation in mice resulted in increased susceptibility to other coronavirus infections, indicating the prevalence of unwanted outcomes for the irisin/TLR3 relationship (113, 114).

## Discussion

**Figure 1:**
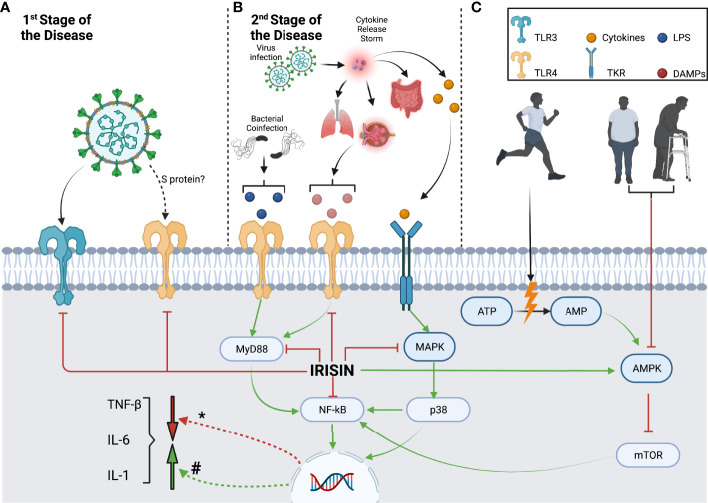
Macrophage inflammatory pathways modulated by irisin during different SARS-CoV2 infection stages and lifestyle contexts. **(A)** The first stage of SARS-CoV2 infection activates innate immune responses through the recognition of PAMPs by TLR3 and TLR4, which are downregulated by irisin. **(B)** The second stage of SARS-CoV2 infection causes cellular damage and induces a cytokine release storm, which activates the MAPK/p38/NF-kB pathway through TKR. CRS results in further organ damage, leading to DAMP release and MyD88/NF-kB pathway activation. Irisin can downregulate both these pathways, decreasing inflammatory cytokine production. In addition, the TLR4 and MyD88/NF-kB pathways can be stimulated by LPS, which can be released due to potential bacterial coinfection. **(C)** Although sedentarism and aging lead to AMPK downregulation and consequent mTOR activation, exercise produces the opposite effect. Exercise-derived AMP and irisin can stimulate AMPK, inhibiting mTOR and downregulating the NF-kB pathway while decreasing cytokine production (#) Cytokine transcription modulation by inflammatory pathways. (*) Cytokine transcription modulation by irisin.

**Figure 1** briefly summarizes the inflammatory pathways that irisin may modulate during SARS-CoV2 infection, while **Table 1** assembles the different effects of COVID-19, irisin, exercise, and sedentarism/aging over the inflammatory pathways discussed in this paper. In the first stage of the disease, SARS-CoV2 stimulates an innate immune response mediated specifically by macrophages, through pathogen-associated molecular patterns associated with TLR3, TLR7, and TLR8. In addition, the SARS-CoV-2 spike glycoprotein binds and activates TLR4 (3). Irisin can reduce TLR3 and TLR4 expression and possibly alleviates the innate immune response in the first stage of the disease, inhibiting the release of proinflammatory cytokines (**Figure 1A**) (29, 86).

**Table 1:** COVID-19, Irisin, Exercise, Sedentarism/Aging, and Obesity influence related inflammatory pathways: TLR4, MyD88, MAPK, AMPK, NF-kB, and cytokine production.

	COVID-19	Irisin	Exercise*
TLR4	Activates (3, 91)	Decreases levels and downregulates its signaling pathway (86, 87)	Decreases levels in the cell surface and mRNA expression (110)
MyD88	Activates (3, 91)	Decreases levels and downregulates its signaling pathway (86, 87)	Decreases levels (108, 109)
MAPK	Activates (65, 66)	Inhibits (63, 64)	Activates signaling pathways responsive to exercise-induced cellular stress (70–72, 75)
AMPK	Activates (115)	Activates (55, 59)	Activates and increases expression (58)
NF-kB	Activates (3, 91)	Inhibits (56, 86)	Increases NF-kB activation through MAPK signaling (73) Downregulates expression through TLR4 signaling downregulation (108, 109)
Proinflammatory cytokine production	Increases (67)	Decreases (6, 56, 59, 63, 64, 86)	Decreases (20, 21, 50, 55)

*We included only studies with regular training exercises of moderate intensity.

In the second stage, the viral infection may result in a cytokine release storm that produces severe systemic inflammation. Increased cytokine levels activate tyrosine kinase receptors in macrophages, inducing MAPK pathway activation (67). In addition, severe systemic inflammation causes organ damage, inducing the production of DAMPs, which further activates the TLR4/MyD88 pathway (88, 90). Both the TLR4/MyD88 and MAPK pathways stimulate NF-kB, inducing an increase in the production of proinflammatory cytokines (**Figure 1B**) (13). Irisin has several modulation targets, including TLR4, MyD88, MAPK, and NF-κB. By reducing their expression levels, irisin reduces proinflammatory cytokine production (**Figure 1B**) (68). In addition, patients with COVID-19 may develop a bacterial coinfection that releases LPS and activates the TLR4/MyD88 pathway (112). Irisin causes a decrease in TLR4 and MyD88 levels, which could prevent the development of sepsis (**Figure 1B**).

Exercise increases energy expenditure and AMP levels, activates AMPK, and suppresses the mTOR pathway (54). On the other hand, sedentarism and aging may have the opposite effects, with a decrease in AMPK activation observed, whereas mTOR activation and proinflammatory cytokine production are induced (**Figure 1C**). Thus, irisin modulation is of particular interest as it counteracts the suppression of the AMPK pathway. In addition, irisin may also ameliorate the proinflammatory state by reducing mTOR and NF-κB activation (**Figure 1C**) (29, 59).

Even though irisin levels may vary following muscle mass, age, and involvement in physical activities, healthy and active people would benefit from irisin’s counterbalance mechanism, considering they would at least present physiological levels. For instance, obese, elderly, and sedentary subjects present loss of muscle mass and consequently lower irisin levels. These groups present a higher risk of developing COVID-19, with a lack of inflammatory counterbalance mechanism. Thus we suggest a potential role of irisin in preventing COVID-19 complications.

One limitation of the present work is that exercise, irisin, and inflammatory status are unveiled. Therefore, further studies have to be made to fulfill the gap in the current knowledge in the field. Furthermore, despite data on irisin and inflammation, COVID-19, and inflammation, the relations between irisin and COVID-19 are still poor. Furthermore, the knowledge about COVID-19 pathophysiology continues to evolve concerning the differences between current and future variants. So further studies will help to understand the role of irisin on COVID-19 outcome. Therefore, this review highlights the potential relation between irisin, inflammation, and COVID-19.

Together, increased irisin levels may counteract the adverse effects of COVID-19 during the various disease stages. Therefore, we hypothesized that irisin could prevent excessive inflammation, perhaps decreasing organ damage and dysfunction and secondary bacterial infection during hospitalization. Irisin’s anti-inflammatory modulation properties may also explain, at least in part, why healthy patients generally have a better prognosis for SARS-CoV2 infection. Further research is needed to understand the role of irisin on COVID-19 patients and may help develop new prevention and treatment strategies.

## Author Contributions

Conceptualization, writing—original draft preparation and editing, and funding acquisition: HA, GL, CF, Gd-A and PB. Final approval: PB and CG. All authors have read and agreed to the published version of the manuscript. All authors contributed to the article and approved the submitted version.

## Funding

This work was supported by grants from Universidade Federal Fluminense (PROPPI/UFF), Coordenação de Aperfeiçoamento de Pessoal de Nível Superior (CAPES) Grant [001], Programa de Biotecnologia da Universidade Federal Fluminense (UFF), Programa de Pós Graduação em Biologia Molecular Celular (UNIRIO), Universidade Federal do Estado do Rio de Janeiro (UNIRIO), Fundação Carlos Chagas Filho de Amparo à Pesquisa do Estado do Rio de Janeiro (FAPERJ) Grants [E-26/010.000983/2019, E-26/203.290/2017, and E-26/2010.592/2019, E-26/201.448/2021], Conselho Nacional de Desenvolvimento Científico e Tecnológico (CNPq), and Instituto Oswaldo Cruz, FIOCRUZ. We also acknowledge financial support by the European Community’s Seventh Framework Programme (FP7-2007-2013) under grant agreement [HEALTH-F4-2011-282095] (TARKINAID).

## Conflict of Interest

The authors declare that the research was conducted in the absence of any commercial or financial relationships that could be construed as a potential conflict of interest.

## Publisher’s Note

All claims expressed in this article are solely those of the authors and do not necessarily represent those of their affiliated organizations, or those of the publisher, the editors and the reviewers. Any product that may be evaluated in this article, or claim that may be made by its manufacturer, is not guaranteed or endorsed by the publisher.
